# Can compassion impact us on a cellular level? Preliminary findings on the effects of a compassion focused intervention on immunological markers and CTRA gene expression

**DOI:** 10.1192/j.eurpsy.2024.696

**Published:** 2024-08-27

**Authors:** M. Matos, P. Rodrigues-Santos, L. Sousa, M. P. Lima, L. Palmeira, A. Galhardo, M. Cunha, I. Albuquerque, P. Gilbert

**Affiliations:** ^1^Faculty of Psychology and Educational Sciences, Center for Research in Neuropsychology and Cognitive and Behavioural Intervention (CINEICC); ^2^Laboratory of Immunology and Oncology, Center for Neuroscience and Cell Biology (CNC); ^3^Center for Innovation in Biomedicine and Biotechnology (CIBB), University of Coimbra; ^4^ Clinical Academic Centre of Coimbra (CACC); ^5^Center of Investigation in Environment, Genetics and Oncobiology (CIMAGO), Faculty of Medicine; ^6^ Coimbra Institute for Clinical and Biomedical Research (iCBR), Faculty of Medicine; ^7^Center for Research in Neuropsychology and Cognitive and Behavioural Intervention (CINEICC), University of Coimbra, Coimbra; ^8^Universidade Portucalense, Porto; ^9^Instituto Superior Miguel Torga, Coimbra, Portugal; ^10^College of Health, Psychology & Social Care, University of Derby, Derby, United Kingdom

## Abstract

**Introduction:**

Addressing mental and physical health problems and promoting wellbeing in educational settings is a global priority. Teachers present a high risk of stress and burnout, which negatively impacts their professional performance as well as their mental and physical health. Compassion-based interventions have been found effective in promoting psychosocial and physiological wellbeing.

**Objectives:**

The current paper presents preliminary findings of the impact of a 6-module Compassionate Mind Training intervention for Teachers (CMT-T) on immunological markers and the Conserved Transcriptional Response to Adversity (CTRA; a gene expression signature that involves a group of 53 genes: pro-inflammatory genes, type I interferon response and genes related to antibody synthesis).

**Methods:**

A pilot non-controlled study was conducted in a sample of public-school teachers in Portugal (*n*=36). Participants were assessed at 4 time-points: 1) Extended Baseline Control_M0, in order to establish a within-subjects psychological and biophysiological baseline (8 weeks before the start of the CMT-T); 2) Pre-intervention_M1 (8-weeks after M0); 3) Post-intervention_M2 (8-weeks after M1); and 4) Follow-up_M3 (3 months after the CMT-T end). In all assessment moments, participants completed a set of psychological self-report measures and were assessed in immunological and epigenetic biological markers through the collection of blood. After M1, teachers completed the 8-week group CMT-T intervention and given access to its resources and materials. They were instructed to practice daily and incorporate the teachings in their personal and professional lives. All assessments and the CMT-T intervention took place at the schools.

**Results:**

Preliminary data on the impact of CMT-T on Immune Response Profiling revealed that teachers’ Natural Killer (i.e., NK) cells were decreased after the CMT-T intervention. In regard to the CTRA gene expression, results showed that type one interferon response genes (e.g., IFI16, IFI27L2, IFITM2, IFITM3, IFITM4P) were decreased after the intervention. In addition, we observed that the gene c-Jun, a pro-inflammatory gene, had a decreased expression after the CMT-T intervention.

**Conclusions:**

These preliminary findings seem to corroborate previous studies involving the type one interferon response, the pro-inflammatory genes and antibody synthesis genes in a signature involving 53 genes previously described as the CTRA gene signature. Furthermore, our results suggest that cultivating compassion using a compassion focused intervention may have a positive impact on markers of the immune system response, associated with how our bodies respond to stress, infection and cancer, as well as, on reducing the expression of genes related to our bodies’ response to stress and inflammation.

**Disclosure of Interest:**

None Declared

